# The relationship between resting metabolic rate and quality of life is moderated by age and body composition in women: a cross-sectional study

**DOI:** 10.1186/s12905-024-03085-0

**Published:** 2024-04-13

**Authors:** Melissa J. Benton, Andrea M. Hutchins

**Affiliations:** 1 Department of Nursing, University of Colorado Colorado Springs, Colorado Springs, CO USA; 2 Department of Human Physiology & Nutrition, University of Colorado Colorado Springs, Colorado Springs, CO USA; 3 Helen & Arthur E. Johnson Beth-El College of Nursing & Health Sciences, University of Colorado Colorado Springs, 1420 Austin Bluffs Parkway, Colorado Springs, CO 80918 USA

**Keywords:** Health-related quality of life, RAND-36, Physical composite score, Mental composite score, Resting metabolic rate, Body composition

## Abstract

**Background:**

Health-related quality of life (HRQOL) is related to body composition, which is also related to resting metabolic rate (RMR). RMR can be increased by exercise and diet interventions that are not dependent on changes in body composition, so a link between RMR and HRQOL may provide interventions that directly improve HRQOL in women.

**Methods:**

One hundred twenty women (median age 63.5 [IQR: 53.0–71.0] years) completed one-time measurement of body composition (multi-frequency bioelectrical impedance), RMR (handheld calorimetry), and HRQOL (RAND-36). Physical (PCS) and mental (MCS) composite scores were calculated for the RAND-36. Pearson correlations were used to identify relationships between RMR, body composition, and HRQOL. Variables at the *p* < .01 level were entered into multiple regression models.

**Results:**

Median body mass index was 26.1 [IQR: 23.2–30.9] kg/m^2^ and median lean mass index was 16.1 [IQR: 14.6–17.3] kg/m^2^. Body composition consisted of fat mass (median 27.2 [IQR: 20.3–34.7] kg) and lean mass (median 42.7 [IQR: 38.2–46.9] kg). Median RMR was 1165.0 [IQR: 1022.5–1380.0] kcal/day. Median HRQOL scores were PCS (84.0 [IQR: 74.0–93.0]) and MCS (85.0 [IQR: 74.3–90.0]). RMR was not directly related to PCS, but was directly and negatively related to MCS (*p* = .002). RMR was significantly and positively related to body composition (lean mass: *p* < .001; fat mass: *p* < .001), body mass index (*p* = .005), and lean mass index (*p* < .001); but only fat mass (PCS: *p* < .001; MCS: *p* < .001) and body mass index (PCS: *p* < .001; MCS: *p* < .001) were related to HRQOL, although the relationship was negative. In addition, age was found to be significantly negatively related to RMR (*p* < .001) and PCS (*p* = .003). Regression models confirmed the moderating influence of age and body composition on the relationship between RMR and HRQOL. RMR, age, fat mass, and body mass index explained 24% (*p* < .001) of variance in PCS; and RMR, fat mass, and body mass index explained 15% (*p* < .001) of variance in MCS.

**Conclusion:**

In women, the relationship between RMR and HRQOL is moderated by age and body composition. Understanding these pathways will allow clinicians and researchers to direct interventions more effectively.

## Background

Health-related quality of life (HRQOL) is negatively influenced by chronic disease, with greater impairments noted as the number of disease diagnoses increases [1]. Furthermore, among those with chronic disease, gender differences exist, and women report significantly poorer HRQOL than men [2–4]. This gender-specific vulnerbility has potentially severe long-term consequences for women because diminished HRQOL is associated with higher all cause mortality [5], mortality due to cancer and heart disease [6, 7], and depression and suicidal ideation [8]. Hence, there is an increasing focus on patient-related outcomes such as HRQOL to improve healthcare processes and outcomes [9–11].

Body composition is also associated with quality of life [12]. Lean (muscle) mass positively influences quality of life [13, 14], while fat mass (obesity) exerts a negative influence [15, 16]. At the same time, body composition, specifically lean mass, positively influences resting metabolic rate (RMR) or the amount of calories burned at rest [17, 18], while a low RMR can negatively affect body composition by promoting increases in fat mass [19, 20] that diminish quality of life [15]. Unfortunately, gender differences are also observed for body composition, with women being at greater risk than men for negative changes that influence and are influenced by RMR (i.e. decreases in muscle and increases in fat) [21].

Resting metabolism can be improved by a variety of exercise and diet interventions that are not dependent on long-term changes in body composition. These include acute bouts of resistance or aerobic exercise [22–24], dietary consumption of protein or essential fatty acids [25, 26], or an overall dietary pattern high in phytochemicals [27]. If a direct link between RMR and HRQOL can be established, this will open up a potentially wide range of interventions that may directly improve quality of life in women. However, we can find no study that has evaluated the direct relationship between RMR and HRQOL, although they are both linked to body composition. Therefore, the current study measured RMR, body composition, and HRQOL in order to identify direct and indirect relationships in women. Our primary hyposthesis was that RMR would be directly and positively related to HRQOL. Our secondary hypothesis regarding body composition was that lean mass would have a positive relationship with both RMR and HRQOL, while fat mass would have a negative relationship with both RMR and HRQOL.

## Methods

### Participants

This was a cross-sectional study. Women who were non-smoking and at least 25 years of age were recruited from the community to complete a single measurement session. Pre-menopausal women were asked to schedule testing 6–10 days after onset of menstruation to avoid any influence of menstrual phase on resting metabolism [28]. All participants were instructed not to eat for at least 4 h, not to drink caffeinated beverages for at least 8 h, and not to exercise for at least 24 h prior to testing to avoid potential influences on RMR [29, 30]. Women who failed to adhere to the study protocol were excluded. A total of 121 women initially enrolled in the study, but one participant was excluded after enrollment for failure to adhere to the study protocol. The study was approved by the University Institutional Review Board (Protocol #19–007) and all participants signed a written informed consent prior to study enrollment.

### Physical activity and health status

Participants were asked to characterize their physical activity level as either *not active, somewhat active, active,* or *very active*. This question has previously been validated for self-report in women, and those who described themselves as active or very active were found to meet the Physical Activity Guidelines for at least 150 min per week of moderate to vigorous physical activity [31]. Health status was assessed using the question, “Has a doctor, nurse, or other health professional EVER told you that you had any of the following?” [32]. Participants were then given a list of common health conditions plus one open-ended “other” option in which they could choose to fill in any health condition not listed.

### Measurements

Hydration status is known to influence body composition and RMR measurement [33–35], so prior to the test session participants were asked to void and normal hydration was confirmed by urine specific gravity using Accutest urine reagent strips (JANT Pharmacal, USA). A range of 1.005–1.030 was considered normal hydration and all participants were within that range at the time of testing. Environmental temperature has been observed to influence RMR, so the room temperature of the laboratory was maintained between 68–77°F (20–25°C) per recommendation [29]. All data were collected by the principal investigators.

#### Anthropometrics

Height was measured to the nearest 0.25 cm using a wall mounted stadiometer. Participants were measured without shoes, with their backs aligned against the measuring rod and heads in the Frankfort horizontal plane. Weight was measured to the nearest 0.1 kg using a portable computerized scale (Tanita, USA). Participants were measured without shoes and wearing only light clothing. Waist circumference was measured to the nearest 0.5 cm with a Gulick tape measure at waist (umbilicus) level. Body weight was normalized to height by calculation of the body mass index (BMI) as weight (kg) ÷ height^2^ (m^2^).

#### Resting metabolic rate

Resting metabolism was measured using handheld calorimetry (MedGem, Microlife, USA). The MedGem is an indirect calorimeter that has been validated and previously used with healthy adults as well as clinical populations [36–41]. The MedGem is not only valid and reliable, but it is also easy to use and minimizes participant burden. Prior to metabolic measurement, participants sat quietly for 10 min. Then, for measurement with the MedGem, a small nose clip was placed over both nares and a mouthpiece was fitted with a firm lip seal. Oxygen consumption was measured continuously for approximately 10 min until the device indicated that the test was complete. RMR was calculated by the device as kcal/day.

#### *Body *composition

Body composition was measured using multi-frequency bioelectrical impedance analysis (Quadscan 4000, Bodystat, Isle of Man). Multi-frequency bioelectrical impedance is a valid and reliable measure of fat and lean mass in adults and compares favorably with standard laboratory measurements using hydrodensitometry (underwater weighing), air displacement plethysmography (BodPod), and dual x-ray absorptiometry (DXA) [42]. Prior to testing, participants rested for 5 min in a supine position with all extremities extended, feet apart, and hands away from their sides. Two electrodes (similar to bandaids) were placed on the right hand and right foot. The test is painless and requires less than 30 s for completion after the initial period of rest. Body composition was calculated by the device as absolute (kg) and relative (%) fat and lean mass. To normalize lean mass between participants, the lean mass index (LMI) was calculated as lean mass (kg) ÷ height^2^ (m^2^).

#### Health-related quality of life

The Rand 36-Item Health Survey (RAND-36) was used to assess HRQOL. The RAND-36 is a 36-item scale that allows separate analysis of 8 subscales for physical and social functioning, physical and emotional role limitations, vitality, emotional well-being, pain, and general health [43, 44]. Subscale scores range from 0–100 and higher scores indicate better quality of life. Unweighted composite scores for physical health (PCS) and mental health (MCS) have been validated for the RAND-36 [45]. Composite scores are calculated by averaging individual subscale scores representing physical health (physical functioning, physical role limitations, pain, general health) and mental health (social functioning, emotional role limitations, vitality, emotional well-being). Composite scores range from 0–100, with higher scores indicating better physical and mental HRQOL.

### Sample size calculation

As no previous data were available for prediction of the direct relationship between RMR and HRQOL, we looked at previous relationships between body composition and RMR reported by Sparti and colleagues [18]. In women, correlation coefficients ranged from 0.61(fat mass) to 0.82 (lean mass). Therefore, sample size was calculated based on a conservative estimate of a moderate correlation of *r* = 0.3 between RMR and HRQOL. To obtain 80% power (β = 0.20) and a two-sided α = 0.01, we estimated that a sample of 120 women was needed.

### Statistical analysis

Data were analyzed using SPSS version 28 (IBM Statistics, USA). The Shapiro–Wilk test was used to evaluate normality. Data were non-normally distributed so participant characteristics were reported as medians (IQR) or frequencies (%). Pearson correlation analysis was used to identify the direction and statistical significance of relationships between the variables of interest, including demographics, anthropometrics, RMR, and body composition. Variables found to have relationships at the *p* < 0.01 level were then considered for entry into multiple regression models to calculate the proportion of variance in HRQOL explained by RMR and body composition.

## Results

One hundred twenty women between the ages of 25 – 89 years completed the study. Participant characteristics are reported in Table 1. The majority (82%) were white, while black and Hispanic women made up 17% of the sample. Sixty-three percent (*n* = 75) characterized themselves as either active or very active. Median waist circumference was 88 cm and median BMI was 26.1 kg/m^2^, indicating borderline health risk [46]. Consistent with this risk, 27% reported hypertension and 18% reported a history of cancer. Although the median value for BMI was in the overweight category, actual values ranged from 17.2 kg/m^2^ (underweight) to 51.9 kg/m^2^ (severe obesity). Values for RMR ranged from 710–2160 kcal/day, with a median of 1165 kcal/day. Finally, median composite HRQOL scores of 84.0 (PCS) and 85.0 (MCS) reflected strongly positive perceptions of physical and mental quality of life.

**Table 1:** Participant characteristics

	**Median (IQR)**	**Number (%)**
Age (years)	63.5 (53.0–71.0)	
Race/Ethnicity		
White		98 (82)
Black		8 (7)
Hispanic		9 (8)
Asian		1 (1)
Hispanic/Black		2 (2)
American Indian		1 (2)
Comorbidities		
Hypertension		32 (27)
Heart disease		8 (7)
Diabetes mellitus		7 (6)
Cancer		22 (18)
Asthma		3 (3)
COVID-19		3 (3)
Physical activity level		
Not active		6 (5)
Somewhat active		39 (33)
Active		54 (45)
Very active		21 (18)
Body weight (kg)	69.4 (63.0–78.8)	
Waist circumference (cm)	88.0 (78.0–95.9)	
Body mass index (kg/m^2^)	26.1 (23.2–30.9)	
Lean mass index (kg/m^2^)	16.1 (14.6–17.3)	
Resting metabolic rate (kcal/d)	1165.0 (1022.5–1380.0)	
Body composition		
Fat mass (kg)	27.2 (20.3–34.7)	
Fat mass (%)	39.6 (34.0–45.3)	
Lean mass (kg)	42.7 (38.2–46.9)	
Lean mass (%)	60.5 (54.8–66.0)	
RAND-36 subscales		
Physical functioning	95.0 (85.0–100.0)	
Physical role limitations	100.0 (75.0–100.0)	
Emotional role limitations	100.0 (67.0–100.0)	
Vitality	65.0 (50.0–80.0)	
Emotional well-being	84.0 (76.0–88.0)	
Social functioning	100.0 (75.0–100.0)	
Pain	80.0 (67.5–90.0)	
General health	80.0 (70.0–90.0)	
Quality of life		
Physical health composite score	84.0 (74.0–93.0)	
Mental health composite score	85.0 (74.3–90.0)	

Correlation analysis demonstrated no significant relationship directly between RMR and physical quality of life (PCS). However, RMR was directly and significantly related to mental quality of life (MCS), but the direction of association was negative (*r* = -0.282, *p* = 0.002). RMR was also significantly and positively related to body composition through absolute (kg) lean and fat mass (*r* = 0.709 and *r* = 0.339, *p* < 0.001, respectively), but only fat mass was significantly related to quality of life (PCS: *r* = -0.329, *p* < 0.001 and MCS: *r* = -0.319, *p* < 0.001), although the direction of the relationship was negative. Furthermore, RMR was strongly and positively related to LMI (*r* = 0.650, *p* < 0.001), but as with lean mass (kg), there was no relationship between LMI and either physical or mental quality of life. Last, RMR was significantly and positively related to BMI (*r* = 0.256, *p* = 0.005), which was in turn significantly and negatively related to both physical and mental quality of life (*r* = -0.366, *p* < 0.001 and *r* = -0.304, *p* < 0.001, respectively).

**Fig. 1:**
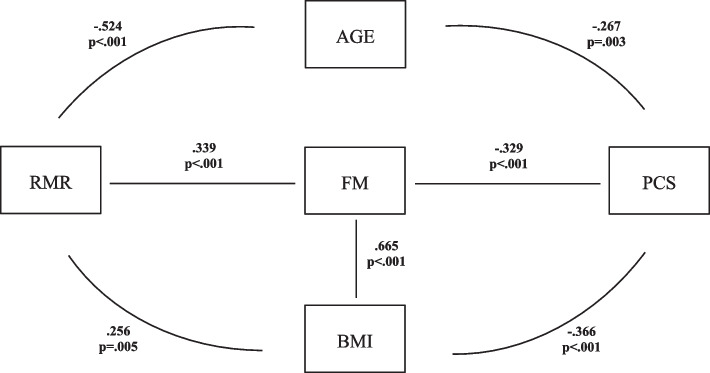
Pathway between resting metabolic rate (RMR) and physical quality of life (PCS) in women. The relationship is moderated by age, fat mass (FM) and body mass index (BMI)

**Fig. 2:**
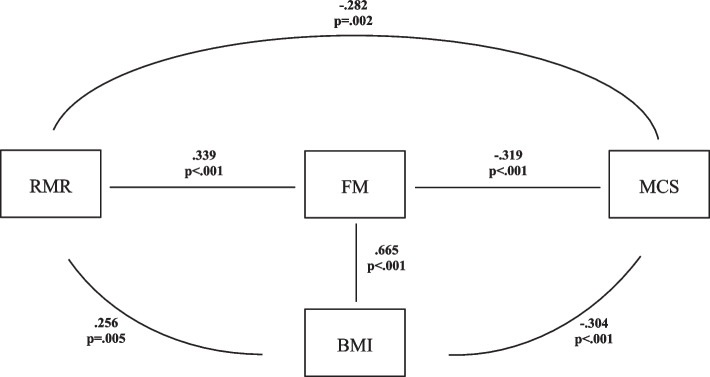
Pathway between resting metabolic rate (RMR) and mental quality of life (MCS) in women. The relationship is moderated by fat mass (FM) and body mass index (BMI)

To further explain the absence of the expected direct and positive relationship between RMR and physical quality of life, we considered demographic variables. Age was found to have a significant and negative relationship to both RMR (*r* = -0.524, *p* < 0.001) and physical quality of life (*r* = -0.267, *p* = 0.003). However, it was not directly related to mental quality of life, or to BMI and fat mass. Explanatory models for HRQOL were developed based on the direction and strength of the relationships between variables (Figs. 1 and 2).

To confirm the moderating influence of age and body composition on the relationship between RMR and HRQOL, we entered them into multiple regression models sequentially based on the strength of the individual relationships. In total, RMR, age, fat mass, and BMI explained 24% (*p* < 0.001) of the variance in physical quality of life (Table 2). In total, RMR, fat mass, and BMI explained 15% (*p* < 0.001) of the variance in mental quality of life (Table 3).

**Table 2:** Multiple regression analysis of the contribution of selected variables to the variance in the physical component (PCS) of HRQOL

PCS	B	95% CI	SE	β	*R*^*2*^	*P*
Model					.24	< .001
Constant	138.18	113.38, 162.98	12.52			
RMR	-.015	-.03, .00	.01	-.22		.044
Age	-.406	-.62, -.19	.11	-.38		< .001
BMI	-.48	-.86, -.11	.19	-.28		.012
FM	-.08	-.43, .27	.18	-.05		.66

*RMR* resting metabolic rate, *BMI* body mass index, *FM* fat mass (kg)

**Table 3:** Multiple regression analysis of the contribution of selected variables to the variance in the mental component (MCS) of HRQOL

MCS	B	95% CI	SE	β	*R*^*2*^	*P*
Model					.15	< .001
Constant	105.48	92.35, 118.61	6.63			
RMR	-.011	-.02, -.001	.01	-.19		.039
BMI	-.24	-.59, .11	.18	-.15		.184
FM	-.21	-.52, .11	.16	-.15		.199

*RMR* resting metabolic rate, *BMI* body mass index, *FM* fat mass (kg)

## Discussion

To our knowledge this is the first study to evaluate the direct relationship between RMR and HRQOL in women. Although the predicted relationship was found with mental quality of life, it was not in the direction hypothesized. Furthermore, no direct relationship was observed with physical quality of life. Hence, our primary hypothesis was not upheld. Our secondary hypothesis was also not fully supported in so far as lean mass was only related to RMR and not to HRQOL. Furthermore, although fat mass was related to both RMR and HRQOL, the direction of the relationship was negative as we hypothesized only for HRQOL. Contrary to our hypothesis, fat mass was positively related to RMR, which we interpret to indicate that as women gain body mass (either fat or lean mass), RMR increases commensurately. This interpretation appears to be supported by the similar relationship observed between RMR and BMI, which is a normalized measure of body weight made up of lean and fat mass.

Our findings regarding the absence of a direct relationship between RMR and physical quality of life were unexpected. Logically, RMR would be expected to have a stronger relationship to the physical component of HRQOL than to the mental component. Physical changes involving loss of lean mass, strength, and function have a well-recognized negative effect on HRQOL [13, 14]. However, these changes are in fact age-related [47], as are decreases in RMR [48]. Although in the current study age was an unexpected moderator for physical HRQOL, Raczkiewicz and colleagues [49] recently reported that in very old women, increased age exerted a negative influence solely on the physical component of quality of life, which provides support for our findings. Further research in this area is needed in order to determine the extent to which age moderates the physical component of HRQOL.

Although the reason why our hypotheses were not supported is unclear, it seems likely that we did not fully appreciate the implications of age. Decreased RMR has been observed with increased age in both women and men across the lifespan [50]. Furthermore, this decrease can exceed what would be predicted based on age-related changes in lean and fat mass, and has not been found to be influenced by participation in physical activity or exercise [51]. In clinical populations such as women with breast cancer, age does not appear to be directly related to HRQOL [52], and instead HRQOL may either increase or decrease with age [53]. Functional ability has been found to be a strong driver of HRQOL in these women [53], and function decreases as age increases [54]. Notably in the current study, age specifically moderated the relationship between RMR and the physical component of HRQOL. Somewhat surprisingly, we can find no study reporting normal changes in HRQOL in healthy women across the lifespan, and instead research seems to have focused specifically on clinical populations [55–57]. Kroenke and colleague [5] previously alluded to this gap in the evidence but subsequent work has not followed. Given the negative and intervening relationship we observed, normal age-related changes in HRQOL should be clarified.

There are other implications of age as a moderator of physical HRQOL. Age may provide a novel strategy by which clinicians can target interventions that increase RMR and potentially improve HRQOL in women. Brief exercise training bouts can produce transient increases in resting metabolism in women in their fifth and sixth decades [22], while dietary consumption of omega-3 fatty acids increases resting metabolism in women in their seventh and eighth decades [26]. Based on the current findings and in recognition of the moderating influence of age, interventions such as these could be directed specifically at older women in order to increase efficiency and clinical effectiveness.

Body composition was also found to moderate the relationship between RMR and both the physical and mental components of HRQOL. Unexpectedly though, only fat mass was found to exert an effect, and although lean mass was strongly related to RMR, the relationship did not extend to HRQOL. Also unexpectedly, fat mass had a positive rather than a negative relationship with RMR, which is contrary to earlier research by Buscemi and colleagues [20]. We anticipated that because a low metabolic rate has been associated with obesity and weight gain [20, 58, 59], women with lower RMR in the current study would demonstrate greater fat mass and likely greater BMI. Mechanistically, a lower RMR could contribute to weight gain since it could be easier to overconsume and push the pathway to kcal storage (i.e., gains in fat mass). Our findings contradicted that, and instead greater accrual of fat mass and body weight (normalized as BMI) was associated with increases in RMR. Body weight or size has been positively associated with increased RMR, but primarily through lean mass [60]. It seems possible that fat mass may also have an effect, especially in the presence of higher body weight manifested as higher BMI. Since fat mass is metabolically active [61], as women gain body fat their RMR may increase since they are adding metabolically active tissue. This would be of particular importance to elucidate given the negative direction of the relationship we observed between body composition and HRQOL.

### Strengths and limitations

In addition to being the first study to evaluate the direct relationship between RMR and HRQOL in women, a strength of the current study is our use of a novel methodology for calculation of unweighted physical and mental health composite scores for HRQOL. These composite scores for the RAND-36 have only recently been validated [45] and their use to date is limited. We believe that their ease and simplicity of calculation will be of interest and use to other researchers using the RAND-36 to assess HRQOL. Unfortunately, the original validation studies for the RAND-36 did not include composite scores to differentiate the physical and mental components of HRQOL [43, 44], which has been a barrier to comparison with the frequently used Medical Outcomes Study SF-36 tool [62].

Our sample size is a potential study limitation. The decision to calculate the current sample size based on previously reported relationships between RMR and body composition may have resulted in under sampling with the potential for a type II error. However, we believe we compensated by setting a pre-determined level of significance of *p* < 0.01. This decreased the possibility of the study findings being due to chance to 1% or less. Therefore, we believe our findings are accurate, if preliminary, and recommend additional future research to further explain the implications of the present findings.

It is also possible that our sample characteristics may have influenced our findings in some way. Although we collected self-reported data regarding comorbidities, we did not collect data regarding medications, some of which may influence RMR or HRQOL. We followed best practice recommendations by limiting consumption of caffeine prior to RMR measurement [29, 30], but we cannot rule out a potentially confounding effect of medications on either measure.

Race and socioeconomic status may also be potential confounders. There are observed differences in RMR between white and black women, with black women having a significantly lower RMR [63]. However, race does not appear to have a significant influence on health-related quality of life in women [64, 65]. Hence, as our sample was predominantly made up of white women, making it relatively homogeneous racially, we do not believe racial differences had a profound effect on our results. Socioeconomic status has a confirmed positive influence on HRQOL, with lower socioeconomic status linked to lower HRQOL in both healthy adults and clinical populations [66, 67]. Ours was a university-based study that recruited widely and randomly from the surrounding community. Although we did not collect socioeconomic data, we believe our sample was representative of our geographic location in the U.S., which has a median household income of approximately $70,000 and a poverty rate of approximately 10%. As with race, we do not believe there was a large degree of socioeconomic disparity within our sample, and for that reason, we do not believe our results were influenced by this factor. In any case, our findings should be considered generalizable only to predominantly white, middle-class women.

Menstrual status may also have influenced our findings. Menopausal status has not been found to be directly related to HRQOL [68], but instead appears to be related to clinical conditions that are themselves related to menstrual status such as osteoporosis [69]. Among actively menstruating women, there is a small effect of menstrual phase on RMR [28]. We attempted to control for potential effects of menstrual phase by asking women who were still menstruating to schedule their test 6–10 days after onset of menstruation. However, we relied on the honor system and did not require confirmation of menstrual status. As menopause has the single most profound effect on menstrual status in adult women and the prevalence of menopause increases with age, it seems likely that any confounding effect of menstrual status would have been manifested through the effect of age that we observed.

Overall, our study sample was homogeneous, which can be considered both a strength and a limitation. We recommend that future research not only extend recruitment to a more racially and socioeconomically diverse sample, but that researchers control for age, menstrual status, and medications in order to elucidate their individual influences.

## Conclusion

In women, the relationship between RMR and HRQOL is moderated by age, fat mass, and BMI. Although RMR has a direct relationship with mental quality of life, the intervening effect of fat mass and BMI is somewhat stronger. By comparison, RMR has no direct effect on physical quality of life and instead, age is negatively related to both. In addition, fat mass and BMI have an intervening influence that is similar to their relationship with mental quality of life. Although these moderating factors were unexpected, understanding the primary and secondary pathways leading to HRQOL in women will allow clinicians and researchers to direct interventions more effectively by targeting specific variables along those pathways, thereby improving health outcomes.

## Data Availability

The data analyzed for the current study are available from the corresponding author on reasonable request.
